# Glaucocalyxin B inhibits cartilage inflammatory injury in rheumatoid arthritis by regulating M1 polarization of synovial macrophages through NF-κB pathway

**DOI:** 10.18632/aging.203567

**Published:** 2021-09-27

**Authors:** Chenyang Han, Yi Yang, Yongjia Sheng, Jin Wang, Xiaohong Zhou, Wenyan Li, Li Guo, Caiqun Zhang, Qiao Ye

**Affiliations:** 1 Department of Pharmacy, The Second Affiliated Hospital of Jiaxing University, Zhejiang, China; 2 Department of Center Laboratory, The Second Affiliated Hospital of Jiaxing University, Zhejiang, China; 3 Department of Rheumatology and Immunology, The Second Affiliated Hospital of Jiaxing University, Zhejiang, China

**Keywords:** Glaucocalyxin B, rheumatoid arthritis, cartilage injury, NF-κB, macrophages

## Abstract

Background: Glaucocalyxin B (Gla B) is a type of sesquiterpenoids. At present, there are rare studies on the pharmacological effects and targets of sesquiterpenoids, while multiple sesquiterpenoids have good anti-inflammatory properties. Therefore, in this study, we aimed to investigate the mechanism of Gla B on macrophages and rheumatoid arthritis.

Methods: LPS/IFN-γ was used to induce M1 polarization of synovial macrophage (SMG) *in vitro*, followed by Gla B pretreatment (5 μM and 15 μM). Afterwards, flow cytometry was performed to detect the proportion of M1 cells (F4/80+CD86+), enzyme-linked immunosorbent assay (ELISA) was used to determine the expression levels of M1 cell markers (TNF-α, IL-1β, IL-6, iNOS and IL-12) as well as M2 cell markers (IL-10 and TGF- β1), immunofluorescence (IF) staining was utilized to measure the expression of CD86, the level of ROS was assessed by probe and Western blot was conducted to detect the expression of P65 and p-P65. M1 polarization was detected in SMG cells with P65 silencing after 15 μM Gla B intervention. The culture medium from M1 cell was used to culture cartilage cells *in vitro*, followed by detection of cartilage cell injury. In animal models, collagen antibodies and LPS were combined to induce RA mouse model. Afterwards, H and E staining was performed to detect pathological changes in mouse joint synovium, safranin O-fast green staining was used to determine cartilage injury, and immunohistochemistry was utilized to detect CD86 and P65 expression. Small molecule-protein docking and co-immunoprecipitation (Co-IP) were used to verify the targeted binding relationship between Gal B and P65.

Results: LPS and IFN-γ could induce M1 polarization in SMG. Gal B could inhibit M1 polarization, decrease the levels of TNF-α, IL-1β, IL-6, iNOS and IL-12, inhibit the expression of P65 and p-P65 while did not affect the expression of IL-10 or TGF-β1. Gal B had no significant effect in SMG cells with P65 silencing. The small molecule-protein docking and Co-IP both showed that Gal B had a targeted binding relationship with P65, and Gal B could inhibit joint injury and inflammation in mice.

Conclusion: Gal B could target the P65 protein. Moreover, Gal B could inhibit the inflammatory injury of articular cartilage in RA by regulating M1 polarization of SMG through inhibiting the NF-κB signaling.

## INTRODUCTION

Rheumatoid arthritis (RA) is an autoimmune disease that involves bones and joints with multi-system inflammation [1]. The pathogenic process of RA involves various different pathways including the innate immune system and the adaptive immune system. Although the pathogenic mechanism of RA remains unclear, massive studies have shown that monocytes/macrophages, neutrophils, etc., are involved in the occurrence and development of RA [2–3]. Macrophages can not only engulf and kill pathogenic microorganisms, but also produce a variety of pro-inflammatory cytokines and chemokines to participate in the pathogenesis of RA [4]. The phenotypes and functions of macrophages are highly heterogeneous and exhibit different phenotypes and functions under the induction of different factors, namely M1 macrophages and M2 macrophages, which is called as the polarization phenomenon of macrophages [5]. During the progression of RA disease, a variety of factors will break the dynamic balance of M1/M2 macrophages, causing imbalanced number and proportion and resulting in an increasing number of M1 pro-inflammatory macrophages, thereby exacerbating the inflammatory response [6].

*Rabdosia japonica* is a plant of the genus Lamiaceae, distributed in Northeast China, North China, Japan, Korea and other regions [7]. *Rabdosia japonica* has a bitter taste, is cold in nature, has the effects of clearing heat and detoxification, invigorating the spleen and promoting blood circulation, anti-tumor and antibacterial inflammation [8]. Pharmacological studies have demonstrated that the terpenoid glaucocalyxin can inhibit the production of platelet activating factor in rabbit platelets induced by carbamicin, inhibit the production of thromboxane A2 induced by arachidonic acid, and increase the level of prostaglandin E2 [9]. Studies have shown that glaucocalyxin can induce apoptosis of HL-60 cells [10]. Glaucocalyxin B is a compound isolated and purified from the *Rabdosia japonica*, without clear anti-inflammatory effect and mechanism. Therefore, in this study, we explored the effect and mechanism of Glaucocalyxin B on synovial macrophage polarization by using RA models.

## MATERIALS AND METHODS

### The inhibitory effect of Gla B on SMG M1 polarization

Mouse synovial macrophage (SMG) (Procell Life Science and Technology Co., Ltd., Wuhan, China) was cultured in complete medium at 37^o^C with 5% CO_2_. After cells reached to the logarithmic phase, cell viability was detected by trypan blue staining. Only cell viability over 80% was further incubated. Cells were divided into DMSO, LPS/IFN-γ (L/I) and Gla B groups. DMSO was used as the control group, and 100 ng/ml LPS (Sigma, USA) and 30 ng/ml recombinant IFN-γ protein (KALANG, USA) was used to induce M1 polarization in L/I group. After pretreatment with 5 μM and 15 μM Gla B for 4 h, LPS/IFN-γ was further added to induce M1 polarization.

(1) Detection of the proportion of F4/80^+^CD86^+^M1 cells by flow cytometry [11]: After LPS/IFN-γ treatment to induce M1 polarization for 48 h, SMG cells were centrifuged, washed with pre-cooled PBS twice, fixed with pre-cooled methanol, incubated with FITC-labeled CD86 monoclonal antibody and PE-labeled F4/80 monoclonal antibody (BD company, Massachusetts, USA) (10 μl) in dark for 20min and washed with PBS twice. The sample was finally resuspended in 50 μl liquid and subjected to flow cytometry. The results were shown in %.

(2) Detection cytokine expression by ELISA: After inducing polarization in SMG for 48 h, the supernatant was collected, centrifuged at 3000 r/min for 20 min and subjected to ELISA according to the manufacturer’s instruction (Abcam, Massachusetts, USA). In brief, the levels of M1 cell markers (including TNF-α, IL-1β, IL-6, iNOS and IL-12) were measured and the levels of M2 cell markers (including IL-10 and TGF-β1) were also detected. The standard curve method was used for calculation and the results were expressed in ng/ml.

(3) Detection of the expression changes of CD86 and P65 by immunofluorescence (IF): SMG cells were inoculated, induced by LPS/IFN-γ for 48 h, washed with PBS for three times, fixed with 4% formaldehyde at room temperature for 0.5 h, permeabilized with 0.2% Triton X-100 for 5 min, incubated with monoclonal antibodies against CD86 and P65 (Abcam, Massachusetts, USA) (dilution 1:300) at 4°C overnight, washed with PBS twice, incubated with fluorescent secondary antibody and mounted with 95% glycerol, followed by observation under fluorescence microscope.

(4) Detect the expression of ROS by DCFH-DA probe [12]: The DCFH-DA probe (Beyotime Biotechnology Co., Ltd., Shanghai, China) was used to detect the intracellular ROS. In brief, cells were seeded into a 6-well plate and subjected to drug intervention for 24 h. The medium was discarded. The DCFH-DA probe was diluted with serum-free medium at a ratio of 1:1000. And 1 ml of medium containing DCFH-DA probe was added to each well, followed by incubation for 30 min. After discarding the medium, cells were washed with serum-free medium twice. The cellular staining level was observed under a fluorescence microscope and the absorbance was detected with a fluorescence spectrophotometer.

(5) Detection of protein expression level by Western-Blot: After LPS/IFN-γ induction for 24 h, cells were collected, washed with pre-cooled PBS and lysed with NP-40 lysate (Beyotime Biotech Co., Ltd., Shanghai, China) on ice for 30 min. After centrifugation, the supernatant was collected and subjected to protein quantification by BCA method (Beyotime Biotechnology Co., Ltd., Shanghai, China). After adjusting protein concentration, the protein sample was subjected to SDS-PAGE electrophoresis, transferred to PVDF membrane, blocked with 5% skimmed milk powder for 2 h, incubated with primary monoclonal antibodies against P65 and p-P65 (Abcam, Massachusetts, USA) diluted in TBST, washed with TBST twice, incubated with horseradish peroxidase-labeled goat anti-rabbit IgG antibody (Abcam, Massachusetts, USA) (dilution 1:2000). After incubation, chemiluminescence method was used for visualization and Image Pro-Plus 6.0 software was used to analyze the optical density. GAPDH was used as the internal control, and the result was expressed as comparison of the optical density value between the target protein and the internal control protein.

### Gla B inhibited SMG M1 polarization through NF-κB (P65)

The primer sequences for shRNA-P65 (pAd1566 plasmid) were as follows: the upstream primer: 5′-CACCGGACTACGACCTGAATGCTGTCGAAACAGCATTCAGGTCGTAGTCC-3′; the downstream primer: 5′-AAAAGGACTACGACCTGAATGCTGTCGAAACAGCATTCAGGTC′. The primers were synthesized by Sangon Biotech Co., Ltd. (Shanghai, China). Cells were divided into L/I, L/I + shRNA-P65 and L/I + Gla B + shRNA-P65 groups. shRNA lentivirus transfection was transfected with Lipofectamine 2000. The level of P65 was detected after transfection for 48 h. Gal B of 15 μM was used. The proportion of M1 type cells and the markers of M1/M2 type cells were detected according to the above method.

### The effect of M1 SMG cells on synovial cell injury

To clarify the injury of M1 SMG on synovial cells, the culture medium from SMG and M1-SMG cells was centrifuged at 3000 r/min for 20 min, which was further used to culture mouse cartilage cells (Procell Life Science and Technology Co., Ltd., Wuhan, China). Control, SMG and M1-SMG groups were set.

(1) Detection of apoptotic level by flow cytometry: Cells were collected, washed with pre-cooled PBS, centrifuged at 3000 rpm/min for 30 min, suspended in binding buffer and staining by using apoptosis detection kit (BD Company, Massachusetts, USA). In brief, cell suspension was incubated with 5 μl of Annexin V-FITC in dark for 5 min, further incubated with 5 μl of PI staining in dark for 5 min, washed with PBS and finally subjected to flow cytometry. Cells with Annexin V-FITC (+) PI (+) and Annexin V-FITC (+) PI (−) were defined as apoptotic cells.

(2) The expression levels of Bcl-2, Bax, and Caspase-3 in cells were detected according to the above method.

(3) The expression level of Caspase-3 in cells was measured according to the above method.

### Gla B inhibited cartilage injury and M1 polarization of macrophages in RA mice

A total of 40 BALB/c mice (8–10 weeks old) were randomly divided into Control, RA and Gla B groups. Mice were intraperitoneally injected with collagen type II monoclonal antibody complex and lipopolysaccharide (LPS) to construct RA model. To be specific, mice were intraperitoneally injected with collagen type II monoclonal antibody complex (5 mg/kg/d) for 10 days. Meanwhile, mice were simultaneously intraperitoneally injected with 100 μg LPS on the first and fourth days. Thus, a stable collagen antibody-induced RA was established in approximately two weeks [13]. Mice in the Gla B group were administered with Gla B (10 μg/ml and 20 μg/ml) by gavage, while mice in the Control and RA groups were treated with equal amounts of normal saline.

(1) Arthritis score measures [14]: During the 10-day period of RA modeling and 5 days of continued culture (15 days in total), arthritis score was measured every 3 days (5 times in total). The macroscopic scoring system was used to evaluate joint inflammation in mice. The criteria were as follows: 11–15 points: the entire feet and toes showed severe arthritis; 6–10 points: more than two joints showed severe arthritis; 1–5 points: two joints showed inflammation; 0 point: no arthritis.

(2) ELISA: Mice were kept for 5 days after model construction. After protein extraction of joint tissue in mice, the expression of TNF-α, IL-1β, IL-6 and iNOS was determined.

(3) H and E staining of mouse joint tissue: The joint tissue was fixed with 4% paraformaldehyde (PFA), dehydrated, transparent, embedded in wax and sliced. According to the conventional experimental procedures of H and E staining, the paraffin-embedded sections were deparaffinized, hydrated, stained, dehydrated, permeabilized and mounted, followed by photography under microscope.

(4) Detection of the expression of CD86 and P65 by immunohistochemistry (IHC): The joint tissue was fixed with 4% PFA, embedded in paraffin, sliced, soaked in 1:50 acetone solution for 3 min, dried, soaked in xylene, treated with gradient ethanol, subjected to antigen retrieval in 0.01 mol/L citrate buffer, treated with 3% hydrogen peroxide for 10–15 min to eliminate endogenous peroxidase, blocked with 5% bovine serum albumin (BSA) at 37°C for 15–30 min, incubated with monoclonal antibodies against CD86 and P65 (Abcam, Massachusetts, USA) at 37°C, incubated with the secondary antibody and visualized with DAB. The slices were counterstained with hematoxylin, soaked in gradient ethanol, dehydrated, transparent and sealed with resin.

(5) Safranin O-fast green staining: The paraffin-embedded slices were deparaffinized, sequentially immersed in xylene I for 20 min, xylene II for 20 min, absolute ethanol I for 5 min, absolute ethanol II for 5 min, 75 % alcohol for 5min and washed with tap water. The slices were stained with Fast green staining for 5–10 min, dried to discard excessive dying solution until the cartilage was colorless, soaked in differentiation solution and washed in tap water. For Safranin staining, the slices were stained with safranin staining solution for 15–30 s and dehydrated quickly with absolute ethanol. The slices were transparent with xylene for 5 minutes, mounted with neutral gum, observed and photographed under microscope.

### Molecule-protein docking model of Gla B and P65

The receptor protein, P65 (PDB ID: 2LWW), was retrieved from the Protein Data Bank database. The following parameters were set: the appropriate box center of P65 receptor protein (center_x = 83.126, center_y = 96.417, center_z = 90.612) and box grid parameters (size_x = 50, Size_y = 60, size_z = 52) to include the active pocket sites that small molecule ligands might bind to. Molecular docking was performed between P65 receptor protein and Gla B ligand small molecules (AutoDock Vina 1.1.2). PyMOL was used to visualize the hydrogen bonding between the receptor protein and the small ligand molecule in three-dimensional images. And Ligplus software was utilized to show the hydrophobic interaction between the receptor protein and the small ligand molecule in two-dimensional images.

### Validation of the targeted-binding relationship between Gla B and P65 by Pull-down assay

In brief, 15μg of recombinant protein was combined with Biotin-labeled Gla B (Biotin-Gla B). Recombinant protein G magnetic beads were incubated with P65 antibody. After washing with Tris buffer, P65 was detected by Western-Blot accordingly, followed by biotin detection by the horseradish peroxidase-conjugated antibiotin antibody (CST, Boston, USA).

### Statistical analysis

The SPSS 20.0 software was used for statistical analysis. The measurement data were expressed as mean ± standard deviation $\left(\bar{x}\;\pm \text{ s}\right)$. One-way ANOVA was used for comparison between multiple groups, and SNK test was used for comparison between groups. A *P* value < 0.05 indicated statistical significance.

### Data availability statement

The data that support the findings of this study are available from the corresponding author upon reasonable request.

### Ethical approval and consent to participate

The study approved with Ethics Committee.

## RESULTS

### Glaucocalyxin B inhibited SMG M1 polarization

**Figure 1:**
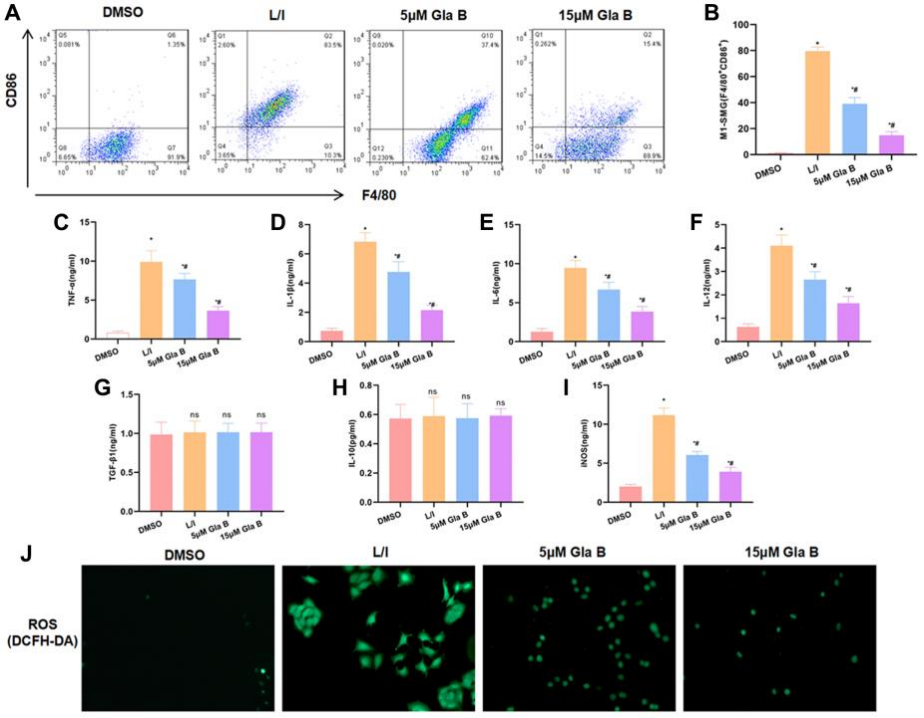
**The effect of Gla B on SMG M1 polarization.** (**A**–**B**) Detection of the ratio of F4/80^+^CD86^+^ M1 type cells by flow cytometry. LPS/IFN-γ could promote SMG M1 type polarization. Gla B could inhibit polarization and decrease the ratio of F4/80^+^CD86^+^ cells. Comparison with DMSO, ^*^P < 0.05; comparison with L/I, ^#^*P* < 0.05. (**C**–**F**) and (**I**) Detection of M1 type cell marker. After LPS and IFN-γ treatment to induce M1 polarization, the levels of TNF-α, IL-1β, IL-6, iNOS and IL-12 were significantly up-regulated, and Gla B could inhibit the up-regulation of cytokines. Comparison with DMSO, ^*^P < 0.05; comparison with L/I, ^#^*P* < 0.05. (**G**–**H**) Detection of M2 type cell marker. After LPS and IFN-γ treatment to induce M1 polarization, the expression of TGF-β1 and IL-10 was not significantly changed, while Gla B also had no effect on the expression of TGF-β1 and IL-10. ^ns^*P* < 0.05. (**J**) Detection of ROS by DCFH-DA. After LPS and IFN-γ treatment to induce M1 polarization, the expression of ROS was significantly up-regulated while Gla B inhibited the expression of ROS.

In our study, F4/80 and CD86 were used to label M1 macrophages. LPS and IFN-γ can induce SMG M1 polarization, with the cell proportion over 80%. Gla B treatment could inhibit M1 polarization and the ratio of F4/80 + CD86 + M1 cells was significantly down-regulated, which was lower than L/I (Figure 1A–1B). The detection of M1 cell marker protein found that the levels of TNF-α, IL-1β, IL-6, iNOS and IL-12 were significantly up-regulated after SMG M1 polarization; and Gla B could inhibit the level of cytokines, which was lower than L/I group (Figure 1C–1F, 1I). The expression of M2 cell marker protein (including TGF-β1 and IL-10) was significantly changed, which was independent of Gla B (Figure 1G–1H). As a marker of M1 cell, ROS was up-regulated in L/I (1.98 ± 0.12), which was significantly higher than DMSO (0.07 ± 0.01); and Gla B could inhibit the level of ROS (1.10 ± 0.55) and (0.44 ± 0.07) (Figure 1J).

**Figure 2:**
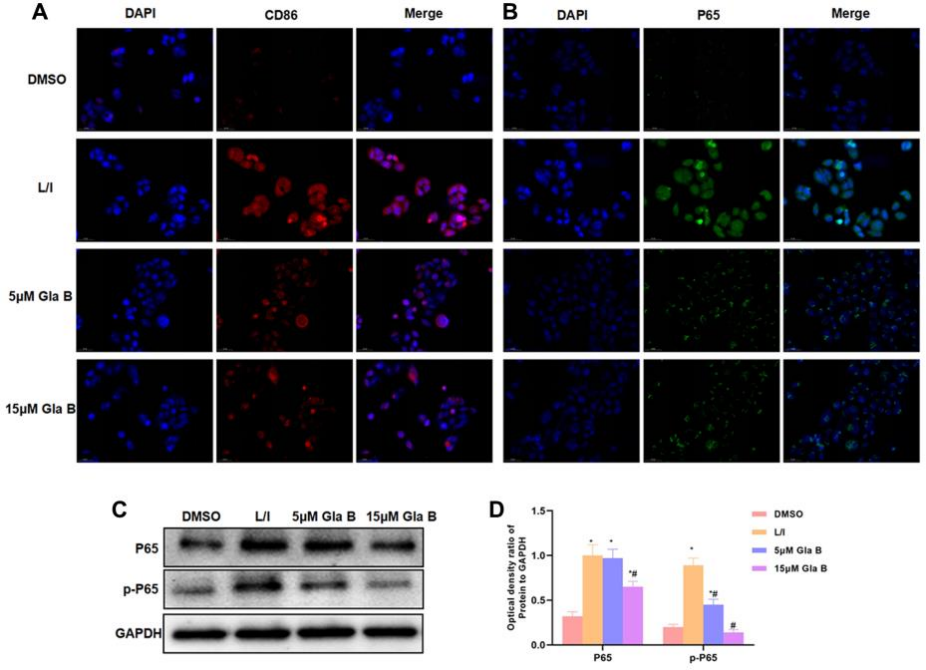
**The effect of Glaucocalyxin B on the expression of CD86 and P65.** (**A**–**B**) IF staining of CD86 and P65 showed that the levels of CD86 and P65 were significantly up-regulated in M1 SMG, which were higher than DMSO. Gla B could inhibit the expression of P65 and CD86. (**C**–**D**) Western-Blot (*n* = 3), the levels of P65 and p-P65 were significantly up-regulated in M1 SMM, which were higher than DMSO. Gla B could inhibit the expression of p-P65 and P65. Comparison with DMSO, ^*^P < 0.05; comparison with L/I, ^#^*P* < 0.05.

IF staining showed that the levels of CD86 and P65 were significantly up-regulated in M1 SMG, which were higher than those in DMSO group. Gla B could inhibit the expression of P65 and CD86, with lower fluorescence intensity than that of L/I group (Figure 2A–2B). Protein detection also revealed that the levels of P65 and p-P65 were up-regulated in M1 SMG, and Gla B could inhibit the expression of P65 and p-P65 (Figure 2C–2D).

### The role of NF-κB (p65) in Glaucocalyxin B-induced inhibition of SMG M1 polarization

**Figure 3:**
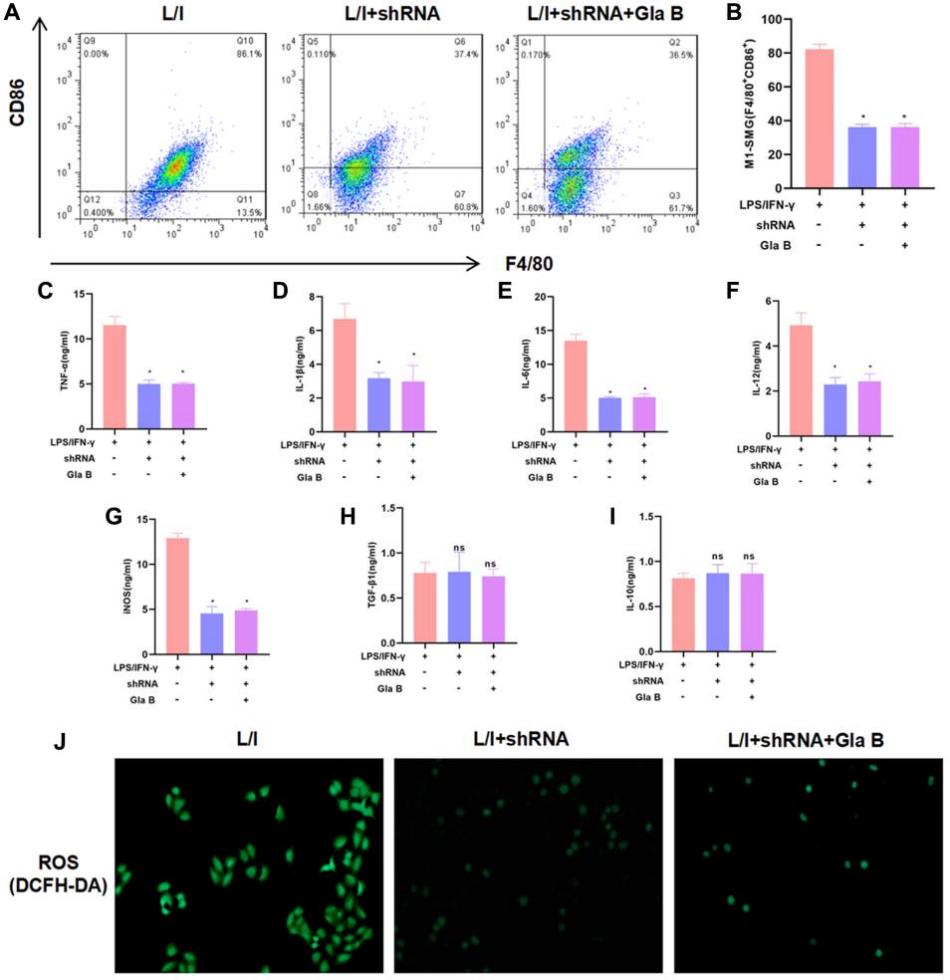
**The role of NF-κB (p65) in Glaucocalyxin B-induced inhibition of SMG M1 polarization.** (**A**–**B**) P65 silencing could inhibit SMG M1 polarization and Gla B cannot further inhibit M1 polarization. There was no difference between groups. Comparison with L/I, ^*^P < 0.05. (**C**–**I**) Detection of M1/M2 cell marker proteins. Gla B cannot further down-regulate the levels of TNF-α, IL-1β, IL-6, iNOS and IL-12 in P65 silenced cells. Comparison with L/I, ^*^P < 0.05. (**J**) Detection of ROS showed that Gla B could not further down-regulate the level of ROS in cells with P65 silencing.

After P65 silencing by shRNA, SMG M1 polarization was induced, followed by the treatment of 15μM Gla B. As a result, P65 silencing could inhibit SMG M1 polarization, which was independent of Gla B. Gla B had no significant effect in shRNA cells, without difference in the proportion of M1 cells (Figure 3A–3B). The detection of cytokines also revealed no significant differences in the levels of TNF-α, IL-1β, IL-6, iNOS and IL-12 between the groups; and no significant difference in the expression of TGF-β1 and IL-10 (Figure 3C–3I). The detection of ROS revealed no significant difference between L/I + shRNA (0.33 ± 0.08) and L/I + shRNA + Gla B (0.31 ± 0.06), which was lower than the L/I group (1.87 ± 0.43), (Figure 3J).

### M1 SMG induced cartilage cell injury

**Figure 4:**
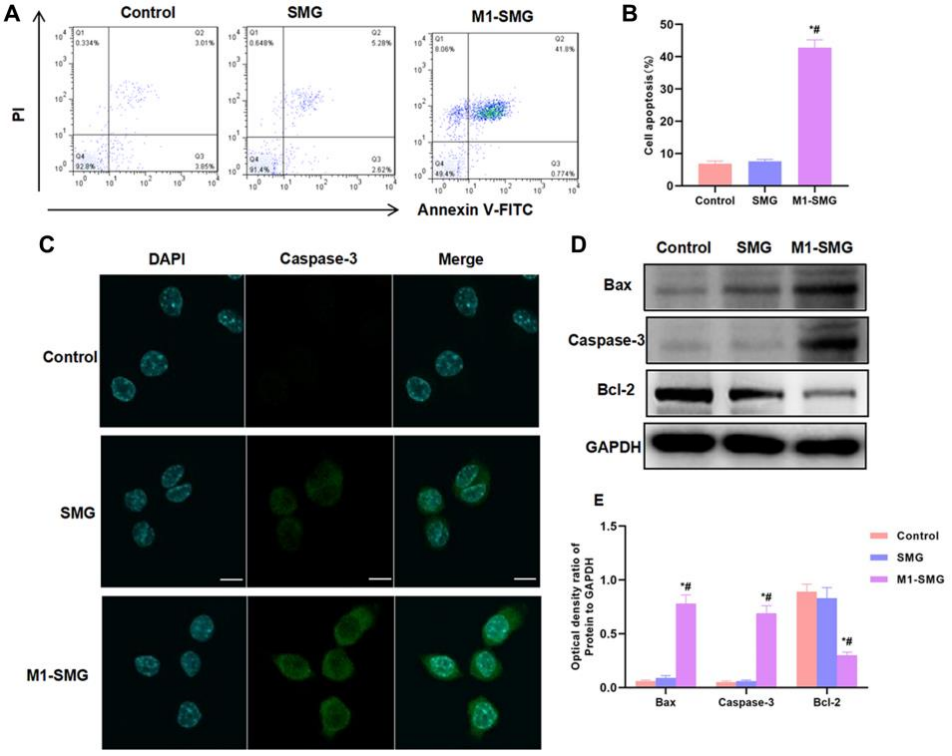
**M1-SMG induced cartilage cell injury.** (**A**–**B**) The results of flow cytometry showed that SMG had no obvious injury to cartilage cells, and the apoptosis rate was not significantly different from that of Control group. M1-SMG promoted apoptosis, with significantly higher apoptotic rate than that of Control and SMG groups. Comparison with Control, ^*^P < 0.05; Comparison with SMG, ^#^*P* < 0.05. (**C**) IF staining showed that there was no significant expression of Caspase-3 in Control or SMG group. The expression of Caspase-3 was up-regulated in M1-SMG, with significantly enhanced fluorescence intensity than that of SMG and Control. (**D**–**E**) Western-Blot (*n* = 3) Detection of protein expression showed that M1-SMG promoted the expression of apoptotic protein (Bax and Caspase-3) and inhibited the expression of Bcl-2. Comparison with Control, ^*^P < 0.05; Comparison with SMG, ^#^*P* < 0.05.

To investigate the possible injury of M1 SMG on cartilage cells, SMG or M1-SMG was co-cultured with cartilage cells. As a result, SMG had no obvious injury on cartilage cells, with a low apoptotic level; M1-SMG could promote cartilage cell injury and increase apoptotic level (Figure 4A–4B). Caspase-3 fluorescent staining showed that Caspase-3 was barely expressed in Control and SMG, while M1-SMG could enhance the expression of Caspase-3 and promote (Figure 4C). Detection of apoptotic proteins also found that M1-SMG promoted the expression of Bax and Caspase-3, with significantly higher relative protein expression level than that of Control and SMG (Figure 4D–4E).

### The effect of Glaucocalyxin B on cartilage injury in RA mice

**Figure 5:**
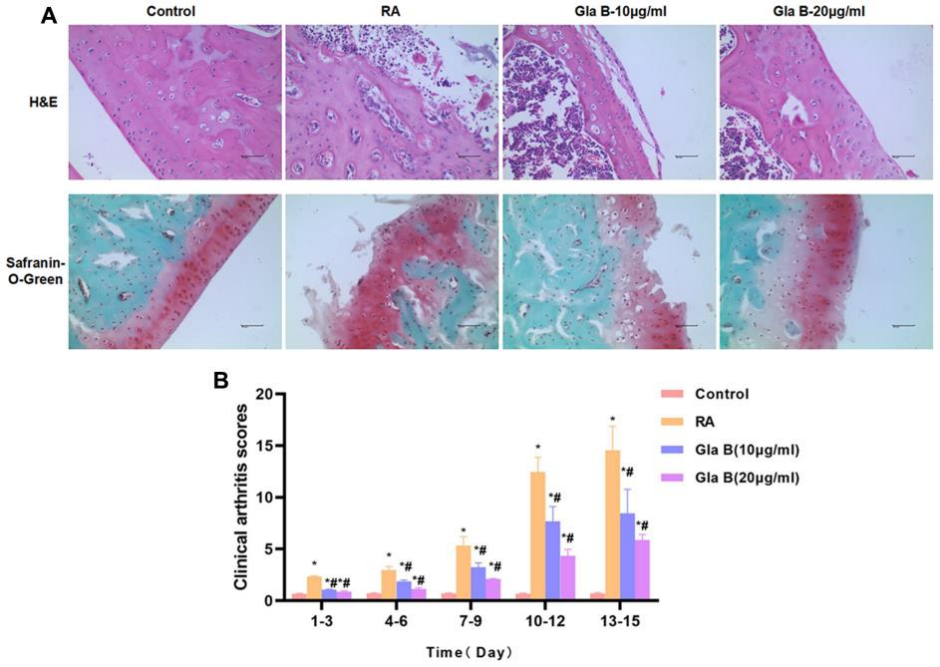
**The effect of Gla B on cartilage injury in RA.** (**A**) H and E and safranin-O-Green staining showed no obvious injury and no inflammatory response of cartilage tissue in Control mice. The cartilage tissue was severely damaged in RA mice, with obvious inflammatory response. Gla B could inhibit the cartilage injury and inhibit the inflammatory response in RA mice. (**B**) The clinical pathological score of RA showed that the score was low in Control group, without obvious pathological change. The score was significantly up-regulated in RA, higher than that in Control group. Gla B could decrease the pathological score. Comparison with Control, ^*^P < 0.05; comparison with RA, ^#^*P* < 0.05.

Collagen antibodies and LPS were combined to construct RA mouse model. The clinicopathological score of RA mice was significantly increased over time, which was significantly different from Control. H and E staining and safranin-O-Green staining showed obvious injury in cartilage, obvious inflammatory response in the cartilage tissue and significant apoptosis, showing abnormal characteristics of cartilage tissue. While the tissue structure was intact in Control group, without no obvious inflammation or injury, which were significantly different from RA mice. Gla B intervention could decrease the pathological score of mice. And the score of the same time period was significantly lower than that of RA (Figure 5B). Gla B could also attenuate cartilage injury in RA mice. The staining results showed that Gla B inhibited cartilage inflammation and bone destruction, which was significantly different from RA in pathology (Figure 5A).

**Figure 6:**
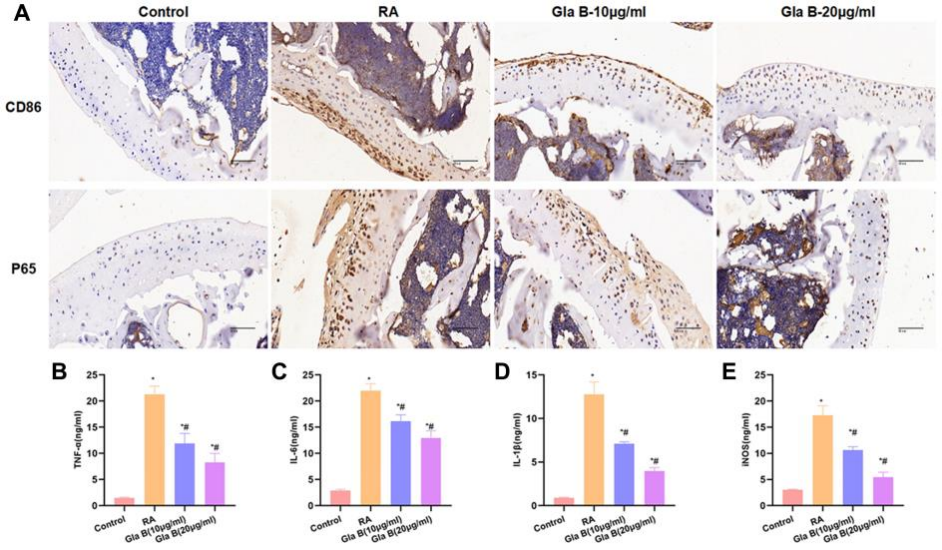
**The effect of Gla B on the expression of M1 type cells and P65 in bone tissue.** (**A**) Detection of CD86 and P65 revealed that the levels of CD86 and P65 were relatively low in Control group, without infiltration of M1 type cells. The expression of CD86 and P65 was significantly up-regulated in RA, with obvious M1 polarization. While Gla B could inhibit the expression of C86 and P65. (**B**–**E**) Detection of M1 type cell markers. The levels of TNF-α, IL-1β, IL-6 and iNOS were significantly higher in RA than those in Control group. Gla B could inhibit the expression of TNF-α, IL-1β, and IL-6 and iNOS, which was significantly decreased compared with RA. Comparison with Control, ^*^P < 0.05; comparison with RA, ^#^*P* < 0.05.

The staining of CD86 and P65 showed no obvious CD86 expression, relatively low infiltration level of M1 cells and relatively low level of P65 in Control group. The expression of CD86 and P65 was significantly up-regulated in RA, which was significantly different from Control group; with obvious infiltration of M1 cells. Gla B could inhibit the levels of CD86 and P65, which was significantly different from RA (Figure 6A). The detection of M1 cell marker protein showed that the levels of TNF-α, IL-1β, IL-6 and iNOS were significantly up-regulated in RA, which were higher than those of Control. Gla B inhibited the expression of TNF-α, IL-1β, IL-6 and iNOS, which was significantly lower than that of RA group (Figure 6B–6E).

### Validation of Glaucocalyxin B targeting P65

Molecular docking revealed a targeted binding relationship between Glaucocalyxin B and the pocket of P65 protein. The binding of Glaucocalyxin B could inhibit the phosphorylation and isomerization of P65, thereby inhibiting the activation of NF-κB. The results showed that Glaucocalyxin B had hydrogen bonds with Glu, Ser and Met. The binding energy was -8.4 kcal/mol. According to the results of docking, Gla B could bind to the protein pocket of P65 to exert the inhibitory effect of P65.

**Figure 7:**
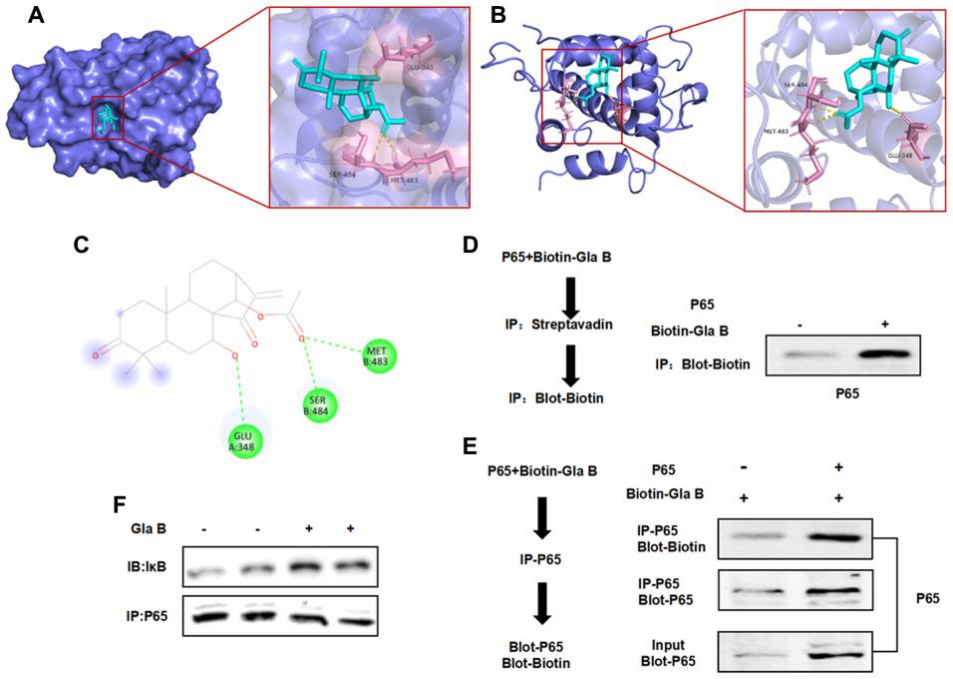
**The targeted binding relationship between Gla B and P65.** (**A**–**C**) 3D and 2D images of the binding between Gla B and P65. Gla B bound to the protein pocket of P65, and Gla B had hydrogen bond with Glu, Ser and Met. The binding energy was −8.4 kcal/mol, which could affect the phosphorylation and isomerization of P65. (**D**–**F**) Pull-down assay showed that Biotin-Gla B had a binding relationship with P65, and Gla B was an effective inhibitor of P65.

Pull-down assay also showed that recombinant P65 protein could bind to Gla B, Biotin-labeled Gla B could bind to P65, with positive expression of Biotin. Taken together, the results of molecular docking and pull-down assay showed that Gla B had a binding relationship with P65 (Figure 7).

## DISCUSSION

The functions of macrophages are highly heterogeneous and plastic. According to the characteristics of surface molecule expression of macrophages, cytokine secretion and arginine metabolism pathway, macrophages can be categorized into M1 and M2 types [15]: M1 macrophages are classically activated macrophages, which are mainly activated by GM-CSF, LPS, etc., and can secrete various pro-inflammatory cytokines (IL-23, TNF-α, IL-6, IL- 1β etc.) [16], with expression of MHC class II, CD80, CD86 and other molecules, can promote the development of inflammation, accelerate the degradation of extracellular matrix and cell apoptosis, and regulate and promote Th1-type immune response [17]. RA is a chronic inflammatory disease, mainly synovitis, and is mainly characterized by inflammation of the joints and infiltration of innate and adaptive immune cells [18], with obvious proliferation of the inner lining layer and subsynovial layer, the proliferation of fibroblasts, the excessive production of pro-inflammatory mediators by macrophages and lymphocytes, such as TNFα, IFNγ, IL-1β, IL-6, IL-17, etc., can promote angiogenesis, lead to the formation of pannus and cause destruction of adjacent of cartilage and bone [19]. Macrophages are involved in various aspects of the inflammatory response of RA, such as stimulating angiogenesis [20], recruiting neutrophils, monocytes and lymphocytes [21], promoting the proliferation of fibroblasts and the secretion of proteases, eventually leading to joint destruction [22]. The synovial macrophages of RA patients show the M1 pro-inflammatory phenotype and have higher expression of pro-inflammatory genes than normal synovial tissues, which can secrete the pro-inflammatory cytokines (IL-1 and TNF-α) to exacerbate the inflammatory response [23]. They can also activate synovial fibroblasts and chondrocytes surrounding the articular cartilage [24] to secrete various proteases, collagenases, matrix degrading enzymes, gelatinase B and leukocyte elastase, etc. [25] to lyse collagen and hyaluronic acid, thereby causing joint tissue destruction [26]. Therefore, how to regulate the polarization of synovial macrophages is one of the important therapeutic approaches of RA [27].

NF-κB is one of the important mediating signals of M1 polarization of macrophages, and the development of effective NF-κB is also the focus of research [28–29]. Glaucocalyxin B is a type of sesquiterpenoid. Handelin chrysanthelide is an effective inhibitor of NF-κB and has certain similarities with Glaucocalyxin B in structure. In this study, we found that LPS and IFN-γ could induce SMG M1 polarization, and SMG had the characteristics of macrophages. This process was accompanied with the high expression of inflammatory factors (including TNF-α, IL-1β, IL-6, iNOS and IL-12). Meanwhile, M1-SMG could also cause cartilage cell injury and promote cartilage cell apoptosis. The treatment of Gla B could significantly inhibit the effects of LPS and IFN-γ, significantly down-regulate the proportion of M1 SMG cells and decrease the expression of TNF-α, IL-1β, IL-6, iNOS and IL-12, indicating that Gla B suppressed the M1 polarization of SMG. As a marker of M1 type cells, ROS was also expressed in M1 SMG, which could be inhibited by Gla B. In addition, we found that Gla B was an inhibitor of P65, could bind to the hydrophobic pocket of the protein and had hydrogen bonding with Glu, Ser and Met. Therefore, pull-down assay also validated the combination of Gla B and P65. In order to further explore the interaction between P65 and Gla B, P65 was silenced in SMG. We found that when P65 was silenced, Gla B failed to further inhibit the M1 polarization of SMG, indicating that Gla B lost its effect after P65 silence, which reversely proved the relationship between P65 and Gla B. Studies on cartilage cell injury have also found no cartilage damage in SMG, while M1-SMG had obvious inflammatory damage, which was associated with the activation of Caspase-3.

In animal models, we also found that RA mice had obvious cartilage damage, accompanied by severe tissue inflammation and apoptosis, which was associated with the infiltration of M1 cells. The expression of CD86 and P65 was significantly up-regulated in RA. P65 is involved in the M1 type polarization and RA injury. Gla B could relieve cartilage injury and inflammatory response in RA, while decrease the expression of CD86 and P65 and suppress the infiltration of M1 cells.

## CONCLUSIONS

In this study, we have found that Glaucocalyxin B, as a small sesquiterpene molecule, can target P65, inhibit the activation of NF-κB signal and the M1 polarization of SMG cells, and simultaneously inhibit the inflammatory response of tissue SMG cells to relieve bone injury of RA, which is one of the mechanisms of action of Glaucocalyxin B against RA.
