# Correction for: Glaucocalyxin B inhibits cartilage inflammatory injury in rheumatoid arthritis by regulating M1 polarization of synovial macrophages through NF-κB pathway

**DOI:** 10.18632/aging.203864

**Published:** 2022-01-31

**Authors:** Chenyang Han, Yi Yang, Yongjia Sheng, Jin Wang, Xiaohong Zhou, Wenyan Li, Li Guo, Caiqun Zhang, Qiao Ye

**Affiliations:** 1 Department of Pharmacy, The Second Affiliated Hospital of Jiaxing University, Zhejiang, China; 2 Department of Center Laboratory, The Second Affiliated Hospital of Jiaxing University, Zhejiang, China; 3 Department of Rheumatology and Immunology, The Second Affiliated Hospital of Jiaxing University, Zhejiang, China

Original article: Aging. 2021; 13:22544–22555. 22544-22555 . https://doi.org/10.18632/aging.203567

**This article has been corrected:** The authors requested a correction for **Figure 3**. The flow cytometry dot plot for the "L/I" group in panel **3A** required correction because the gate was not unified during the flow cytometry. This was corrected by reprocessing the initial data, which did not affect the final quantification. The image for the "L/I+shRNA+Gla B" group in **Figure 3J** resembles the image for the 15 μM Gla group in **Figure 1J.** To avoid confusion, the authors revised **Figure 3J** by using representative images from the original set of experiments. These alterations do not affect the results or conclusions of this work. The authors would like to apologize for any inconvenience caused.

New **Figure 3** is presented below.

**Figure 3:**
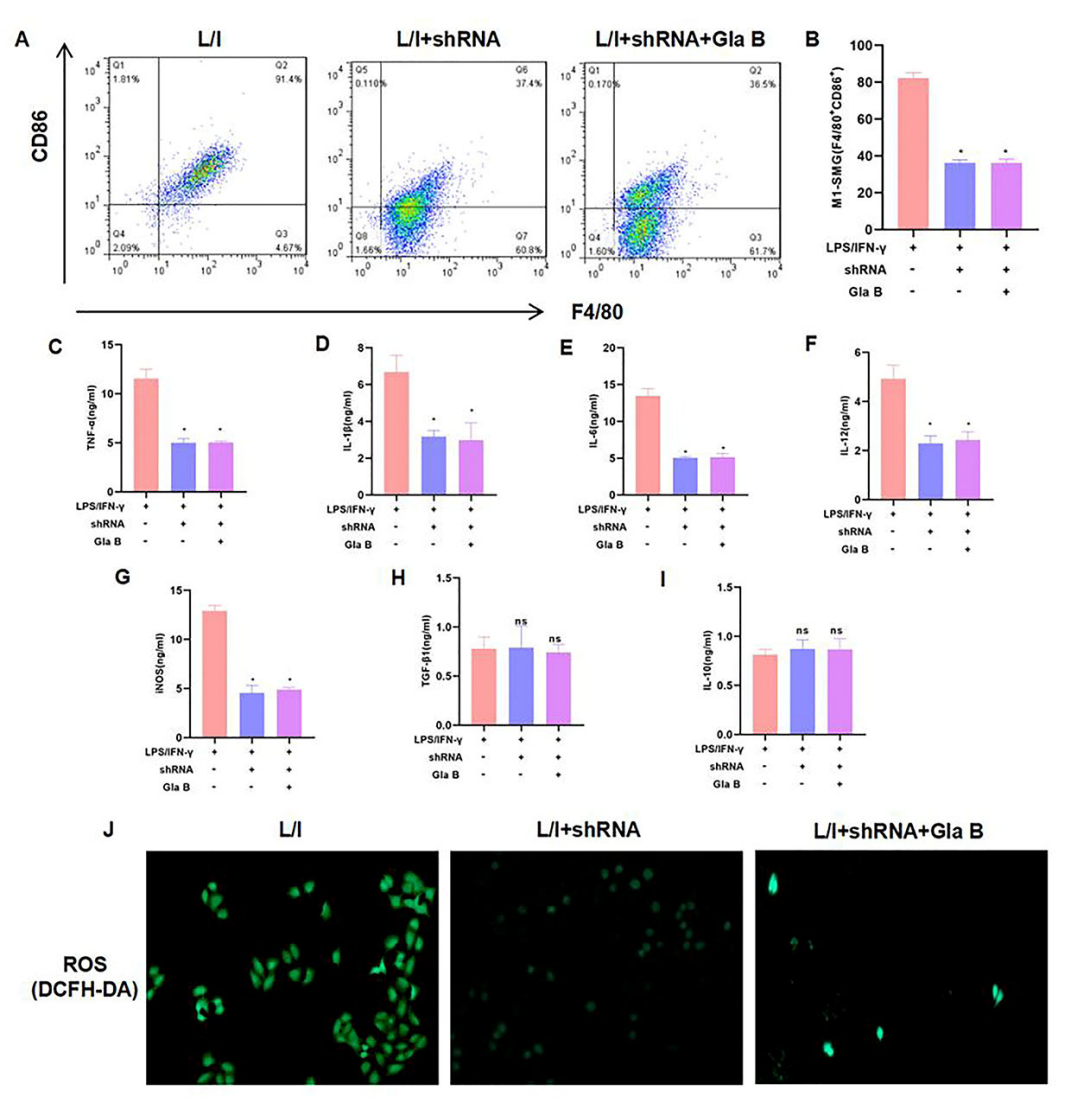
**The role of NF-κB (p65) in Glaucocalyxin B-induced inhibition of SMG M1 polarization.** (**A**–**B**) P65 silencing could inhibit SMG M1 polarization and Gla B cannot further inhibit M1 polarization. There was no difference between groups. Comparison with L/I, *P < 0.05. (**C**–**I**) Detection of M1/M2 cell marker proteins. Gla B cannot further down-regulate the levels of TNF-α, IL-1β, IL-6, iNOS and IL-12 in P65 silenced cells. Comparison with L/I, *P < 0.05. (**J**) Detection of ROS showed that Gla B could not further down-regulate the level of ROS in cells with P65 silencing.

